# Identifying the learning objectives of clinical clerkship in community health in Japan: Focus group

**DOI:** 10.1002/jgf2.289

**Published:** 2019-12-13

**Authors:** Daisuke Kato, Hideki Wakabayashi, Akiteru Takamura, Yousuke C. Takemura

**Affiliations:** ^1^ Department of Family Medicine Mie University Graduate School of Medicine Mie Japan; ^2^ Department of Community Medicine Kameyama, Mie University School of Medicine Mie Japan; ^3^ Department of Medical Education Kanazawa Medical University Ishikawa Japan; ^4^ Department of Family Medicine Graduate School of Medical and Dental Sciences Tokyo Medical and Dental University Tokyo Japan

**Keywords:** clinical clerkship, community‐based medical education, Japan, learning objectives

## Abstract

**Background:**

The value of medical education in the community has been increasingly and globally recognized. In 2015, the World Federation for Medical Education emphasized the importance of medical education in various settings in their standard. Similarly, in Japan, the Model Core Curriculum for Medical Education in Japan (MCCMEJ) is revised in 2016. However, both the learning objectives of such clerkships and their concrete strategies in Japan are not clearly established. In this study, the authors identified the learning objectives of clinical clerkship in community health reflecting the perspectives of medical professionals and community inhabitants.

**Methods:**

They held six focus groups that included physicians, other medical professionals, and inhabitants (n = 35) who were involved in a clinical clerkship in community health at three prefectures in Japan from 2017 to 2018. Further, they recorded, transcribed, and thematically analyzed the discussion using MCCMEJ as conceptual frameworks.

**Results:**

The learning objectives comprised of 13 domains. The following four domains were not found in “Basic Qualities and Capacities for Physicians” in MCCMEJ: “future‐oriented systematic view,” “organic integration of knowledge/skill,” “understanding of the community,” and “awareness as an individual physician.”

**Conclusion:**

With the community inhabitants' participation, the study results reflect the community needs in Japan. The authors hope that the outcome of this study will be useful to further improve clinical clerkship in community health.

## 1 INTRODUCTION

A community‐based medical education for undergraduate medical students is highly recommended globally because there are more needs in the community than in the tertiary hospitals.^1^, ^2^, ^3^, ^4^ In Japan, also, the standards were published in 2015, which was conformable to the global standard by the World Federation for Medical Education (WFME).^5^ There, the clinical clerkship in community health (CCC) is required. The Model Core Curriculum for Medical Education in Japan (MCCMEJ), revised in 2016 by the Ministry of Education, Culture, Sports, Science, and Technology (MEXT), expected the more enrichment of CCC.

It is an urgent issue to develop the CCC curriculum in Japan. However, it is not easy to establish learning objectives in CCC. The concrete goals differ according to the communities and medical schools^6^ because medical education programs and communities are associated in numerous ways.^6^ Harden suggested educational strategies in curriculum development, the SPICES model,^7^ based on community medicine. It is important to establish such national‐level, not community‐level, learning objectives in CCC in terms of quality management of physicians working in communities.

No studies have reflected the perspectives of community inhabitants in Japan to establish learning objectives in CCC. Community involvement is expected to provide better learning to medical students.^8^ Therefore, it is essential to gather opinions from stakeholders not only in a university but also in a community (eg, medical professionals and inhabitants there).

Considering the overseas cases, one of the examples of CCC is longitudinal integrated clerkship (LIC). LIC is featured in its continuity.^9^ More than half of the LIC is longer than 6 months.^10^ Because LIC is expected to resolve the shortage of physicians, it is sometimes conducted in a rural area.^10^ We will not be able to adapt the learning objectives directly into medical education in Japan because of its settings: length and location.

In this study, we aimed to establish the learning objectives for undergraduate medical students in CCC through focus groups with medical professionals and inhabitants in the community and to compare the conventional and overseas ones.

We used MCCMEJ as the conceptual framework for this study. MCCMEJ established by MEXT has been playing the central role in medical education in Japan, and the study result based on MCCMEJ would, therefore, be more effective and familiar to medical professionals including physicians involved with CCC.

## 2 MATERIALS AND METHODS

Between November 2017 and March 2018, we conducted a focus group six times in Ishikawa, Kochi, and Mie prefectures. These prefectures are separated regionally with each other and covering from mountain to sea areas, and from urban to rural areas. Universities in these prefectures have achieved a higher quality in CCC both quantitatively and qualitatively, in terms of the number of medical students they sent to communities, the contents of the practice, and the length of CCC. Among these universities, CCC is practiced by the Department of Family Medicine and its corresponding institution.

Regarding the participants' recruitment in the focus group, we carried out purposive sampling. We asked the CCC program executives in the university to choose research participants based on the inclusion criteria. The recruitment by the program executives would be reasonable and practical because of their knowledge, experience, and human relationship in the community.

For the recruitment of medical professionals, the inclusion criteria included individuals who belonged to the community and/or university and were involved in medical education through CCC. For the recruitment of inhabitants in a community, the inclusion criteria included patients, patient's family, and stakeholders (eg, community managers and welfare commissioners).

Through six‐time focus groups, the discussion targeted 35 medical professionals and community inhabitants (Table S1). First, the research participants in each community were divided into two groups: physicians alone and other medical professionals and inhabitants. By forming a group with similar backgrounds in the focus group, we acquired less affected data by the authority gradient among members.^11^ By adding to the inhabitants' group the medical professionals except for physicians, inhabitants expressed their thoughts easily, compared to the focus group with physician interviewers only.

For each group with 5‐10 research participants, we discussed the learning objectives for 1 hour that should be achieved by medical students before graduation. Two interviewers conducted the focus group (the main facilitator [DK] and the subinterviewer [HW]) in accordance with the interview guide (Table 1). At the end of each discussion, the main interviewer summarized the discussion to ensure that all research participants in each focus group were able to express their thoughts adequately, and all members confirmed that no other comments and differences in terms of content were observed. Recorded discussions were transcribed verbatim by a professional company, and we analyzed the transcribed data with thematic analysis.^12^ Specifically, the main interviewer generated codes from the data and assembled several codes into domains. Data from medical professionals and from community inhabitants were analyzed separately. The analyzed results were confirmed by all authors, and differences were discussed until consent was obtained from all authors. The authors who analyzed the data were family physicians.

**Table 1:** Interview guide

What are the physician's competencies in community medicine? What are the physician's roles in community medicine? What are the learning objectives that should be achieved by medical students through clinical clerkship in community health before graduation? Why?

This study was approved by the ethics review board of Mie University (approval no. 1771). The aim of the research was explained to the medical professionals and inhabitants. All participants signed a form indicating their consent to participate.

## 3 RESULTS

With MCCMEJ as a conceptual framework, we identified the learning objectives of CCC consisted of 13 domains. They covered the full of nine domains in “Basic Qualities and Capacities for Physicians” in MCCMEJ (Table 2), which shared the learning objectives for junior residents for the seamless medical education from undergraduates to postgraduates.^13^ The common nine domains were the following: “professionalism,” “medical knowledge and ability to solve problems,” “medical skills and patient care,” “communication skills,” “team‐based care,” “quality and safety management in medical care,” “medical care in society,” “scientific inquiry,” and “lifelong learning attitude” (Table S2).

**Table 2:** Learning objectives

Professionalism	Have high ethical standards Have a patient‐centered perspective Understand true patients' true reason Build a relationship of trust between inhabitants and students
Medical knowledge and ability to solve problems	Have a wide range of knowledge and experience that enable students to understand the patients' thoughts and values. Treat common diseases Deal with rare diseases Deal with emergency diseases Continue chronic illness treatment Make correct diagnosis Assess basic human functions (eg, diet, exercise, excretion) Ability to adequately deal with other problems Have a viewpoint of prevention Extract and formulate issues using EBM framework
Medical skills and patient care	See not only diseases but also the patient as a whole See patients including their families Know the patient (family composition, presence or absence of social network/relatives, and economic conditions) Have both biomedical and social perspectives Understand the community‐based integrated care systems and anticipate and respond to the patients' future in the sequence of hospitalization, discharge, and ambulation See multimorbidity
Communication skills	Communicate with patients and their families Explain the patients' condition briefly and easily
Team‐based care	Understand the role of any kind of medical profession Understand the role of local government Understand the welfare system Collaborate with other medical professions
Quality and safety management in medicinal care	Practice resilience
Medical care in society	Understand the community characteristics, needs, issues, and resources and set goals accordingly Understand that physicians are members of the community and should utilize themselves in the community Objectively assess their own medical care level, collaborate with nearby hospitals/clinics, and appropriately refer to specialists Demonstrate leadership in the community Understand the importance of social capital and see inhabitants in the community Understand that there is no interruption between community and other medical facilities (eg, University hospital) Share the goals (eg, health promotion, prevention, welfare) with all kinds of medical professions, inhabitants, and the local government
Scientific inquiry	Conduct a community diagnosis Understand statistical methods and evaluate data objectively To nurture a research mind and conduct a research, such as action research
Lifelong learning attitude	Enhance lifelong learning skill Reflection including the multidisciplinary view Do not focus on outcomes. Understand the importance of the process of experiencing and learning
Future‐oriented systematic view	Understand the existence and role of community medicine Have a comprehensive perspective Explain the value of community medicine with evidence Learn new technologies and concepts such as AI and economy sharing and work with technologies with limited medical resources Understand that there is medical care that can be achieved only by humans Have a work‐life balance perspective Understand the further importance of community medicine in the aged society
Organic integration of knowledge/skills	Learn about unfragmented medical care and how learning so far is used in the real practice Understand the bedside perspective Find a role model
Understanding of the community	Interested in the community Aware of cultures, customs, lifestyles, and values in the community and utilize them in the community medicine
Awareness as an individual physician	Adequate greeting Time compliance Well‐dressed Collaborate with senior physicians Establish a good human relationship with inhabitants

In addition to the above nine domains, we newly identified four domains: “future‐oriented systematic view,” “organic integration of knowledge/skill,” “understanding of the community,” and “awareness as an individual physician” (Table 2).

### 3.1 Future‐oriented systematic view

This domain means to know the existence and roles of community medicine and to have a general practice/family medicine viewpoint. Regarding limited medical resources, the use of new technologies and concepts, such as artificial intelligence (AI) and economy sharing, is required which would improve medical care. Moreover, it would be useful for a work‐life balance.

…I think medical students need to interact with community inhabitants not taking medical care and understand the community through the interaction. (E43)

Medical students are interested in the role of physicians in the community, and I found the word ‘trusting’ frequently in their reports … The existence of medical care based on such a trusting relationship seems to be very fresh to students. (E160)

Such clerkship would provide medical students with some tips regarding learning medical care in the future. There would be a good notice for inhabitants' value, and nice suggestions for medical care. In such a context, AI would be essential. (F89)

The perspective of work‐life balance is important. It is also important to consider how to collaborate together with machines. (F86)

### 3.2 Organic integration of knowledge/skill

This domain emphasizes the importance of real practice based on the knowledge and skills which medical students learned in a classroom ever. This also suggests finding good role models.

When I teach students community medicine, it is important to relate the knowledge they have learned before in the university to community medicine. (E121)

### 3.3 Understanding of the community

This domain means an interest in the community itself and experiences with its culture, customs, lifestyle, and values and learning and coordination with inhabitants.

The most important ability which is especially demanded by communities is to absorb the characteristics of each community. We need the ability to understand cultures, lifestyles, and values in the community. (C14)

### 3.4 Awareness as an individual physician

This domain means the behaviors, which are essential to work as a physician, such as greetings, well‐dressed, and time compliance. This also emphasizes well‐cooperation with senior physicians as well.

Medical students should learn their basic attitude as members of society. It is very important to act appropriately as a member of society because once medical students go out into the community, they become deeply involved in society. Greetings, time compliance, and self‐awareness of social accountability should be included in learning objectives. It is important for medical students to be aware of the limitations of their abilities, appropriately referral them to specialists, and collaborate with other medical institutions. (C41)

## 4 DISCUSSION

In this study, we identified qualitatively the learning objectives of CCC for undergraduates in Japan. To achieve seamless medical education, we adapted Japanese paper (MCCMEJ) as a conceptual framework in this study. We newly identified four domains out of 13. Because the study results reflected the Japanese context and the community inhabitants' participation, they would be more practical and useful.

We did not find the domain regarding the learning about other specialty than general practice/family medicine, while, according to a previous publication, the learning objectives of CCC were categorized into four groups: to learn about general practice/family medicine, to learn about a particular specialty other than general practice/family medicine, to learn about primary care, and to learn multiple disciplines concurrently.^14^ This result would be due to a specific feature of primary care in a community in Japan. Primary care has been delivered by semigeneralist/specialists, that is, physicians who leave hospitals work as GPs in a community without further training or certification.^15^

We extracted the learning objectives regarding the collaboration with community inhabitants. According to the previous review in the United States, few papers pointed out this topic because more attention was paid to the collaboration with healthcare providers.^16^ The difference in the result would be because we gathered the data from community inhabitants. While medical education should reflect community needs,^2^ some papers pointed out the mismatch between them.^17^, ^18^

Concerning “organic integration of knowledge/skill,” medical students, through CCC, need to integrate the knowledge/skill which they gained ever, and this domain related to “Integrated” in SPICES model, also consistent with the WFME standard^5^ and MCCMEJ. Both of these standards required the participation in CCC by the collaboration with relevant organizations, including local government. The community is a field where the medical students integrate their knowledge and skills into reality, which would be unique in CCC. Therefore, this should be classified in a different domain from “Medical knowledge and ability to solve problems” which focuses on knowledge and technology themselves.

Regarding “future‐oriented systematic view,” it is critical to reflect the needs of future medical care on medical education.^2^ Communities in Japan have been encountering many changes such as rapid aging and workforce shortage. New technologies like AI are expected to overcome these challenges from the view of sustainability. Moreover, these learning objectives extend beyond continuing professional development (CPD), which aims to provide better medical care to patients.^14^ Topics such as work‐life balance and the sharing economy showed an emphasis on physicians and communities, as well. That is, interviewees thought this physicians' effort from a broader perspective, not just CPD.

In terms of “understanding of the community,” several papers pointed out the importance to understand the community itself, including areas other than medical care.^19^ Hospital‐based education is sometimes criticized as “ivory tower,”^7^ and we expect such understanding would nurture the community orientation, as well.

About “awareness as an individual physician,” greeting, well‐dressed, and time compliance are critical in CCC because medical students are at the center of medical education in the community involving patients, physicians, communities, and local governments.^20^ CCC is significantly different from conventional medical education. Although they are difficult to define compared with others,^21^ we should include this domain in the learning objectives. Regarding professionalism, it has three learning objectives in the Model Core Curriculum: (a) learn the history of medicine, (b) explain the ethical issues, and (c) explain the norms such as the Hippocratic Oath, the Geneva Declaration, and the physician verification.^13^ The learning objectives we identified do not fall under any of these topics. Further, through CCC, medical students are exposed to the nonmedical aspects of the community. In such a clinical clerkship, it is not appropriate to limit their roles to “future physicians.” Because social behavior is also required in nonmedical contexts, we needed to distinguish this domain from “professionalism,” which is about physicians. This is the same as creating the “Understanding of the community” domain because it is important to understand the community, including nonmedical aspects. It is emphasized in this domain that the scope of CCC should not be limited to “medical care.” These newly created domains of learning objectives illustrate the multifaceted aspects of CCC.

The learning objectives we identified in this study would be useful in CCC in Japan. Such national‐level, not community‐level, learning objectives would contribute to the seamless medical education from undergraduates to postgraduates. The results of this research also related to the Postgraduate Medical Education WFME Global Standards.^5^ This is also important from the view of continuing medical education.^22^

We expect that these learning objectives are consistent with the other prefectures and we conducted sufficient focus groups qualitatively and quantitatively because we got similar learning objectives in the three prefectures. Although the number of prefectures we held the focus group was three, the prefectures were varied in terms of areas and clinical settings in which the CCC program was conducted. Regarding the transferability, the learning objectives should be evaluated by another study method in the future.

There is another limitation in this study. Medical students did not participate in the focus group in this study. We hope to reflect the medical students' opinion on CCC curriculum development.

Despite the limitations above, we think this study has proper strengths. To our knowledge, this is the first study in Japan to identify learning objectives in CCC reflecting the perspectives of medical professionals and community inhabitants. It is important to establish the national‐level learning objectives in terms of quality management of physicians.

Further, this study result reflected not only community needs but also medical education theory. Previous reports revealed that medical professionals in the community require academic advice from the staff of universities.^23^ We included the physicians to the focus group, who belonged to the university and provided medical education to undergraduates.

We identified the learning objectives of CCC in Japan. Four domains out of 13 were newly identified. This study result reflected the Japanese context and community inhabitants' participation in the study. That is, in CCC, it is important not to limit the scope of clinical clerkship to “medical care” and not to limit the role of medical students to “future physicians.” We hope that this result will contribute to the improvement of CCC in Japan and other countries.

## 5 CONCLUSIONS

This study clarified the learning objectives for undergraduate medical students in CCC in Japan. Some of them were not included in MCCMEJ. They were thought to be specific in CCC as well as consistent with the international stream of CBME. We deepened the understanding of the learning objectives of CCC with the SPICES model. We hope that this result will contribute to the improvement of CCC in Japan and other countries.

## CONFLICT OF INTEREST

The authors have stated explicitly that there are no conflicts of interest in connection with this article.
