# Supplementary material for: Identifying the learning objectives of clinical clerkship in community health in Japan: Focus group
Source: J Gen Fam Med. 2019 Dec 13;21(2):3–8. doi: 10.1002/jgf2.289 (PMC7060287; doi:10.1002/jgf2.289)
Supplement: Supplementary file 1 [file JGF2-21-3-s001.docx]

|  | Physicians | Medical professionals | Inhabitants |
| --- | --- | --- | --- |
| A | 6 (6) | 3 (0), 3 (0) | 8 (6) |
| B | 4 (3) | 4 (1) | 1 (1) |
| C | 6 (6) | - | - |
