# Supplementary material for: Identifying the learning objectives of clinical clerkship in community health in Japan: Focus group
Source: J Gen Fam Med. 2019 Dec 13;21(2):3–8. doi: 10.1002/jgf2.289 (PMC7060287; doi:10.1002/jgf2.289)
Supplement: Supplementary file 2 [file JGF2-21-3-s002.docx]

| 1. Professionalism |
| --- |
| - In addition to knowledge and skills, attitude is an important element of medical education. I think attitude would be most important and attitude includes the cooperation, the ability to solve problems through self-study, a sense of mission and ethics. (E -59) - In summary, patient-centeredness is important. (A-157) - Without a relationship of trust with a physician, it is difficult for a patient to talk frankly with the physician...... Unless there is such a relationship, physicians cannot understand the true feelings and medical problems which patients have. (B -44) |
| 2. Medical knowledge and ability to solve problems |
| - Since there are many common diseases in the community, it is necessary to be able to treat them. In addition to common disease, physicians working in the community must also have the ability to triage rare diseases. It is important to have the ability to continuously see patients with chronic diseases.....(E-39) - Physicians working in the community need to have a wide range of knowledge (not limited in medicine) in order to empathize with the various thoughts that patients have. (A -24) - It is important to be able to evaluate the basic function (Diet, exercise, and excretion) of the human. The assessment can be combined with existing medical knowledge to determine the problems and illness in which the patients have. (D -55) - Physicians should learn emergency medicine skills. (B -173) - When working in university hospital, I was asked the evidence in my medical treatment. In the community, I have less opportunities because the number of physicians working at the same hospital is reduced. The ability to review their own medical care based on EBM is necessary. (C -65) - From the view of local government, it is important to work with physicians on health promotion, prevention and welfare. (F -75) |
| 3. Medical skills and patient care |
| - It is important to understand the living conditions of patients. If the patient lives alone, does he or she have a close relative or someone whom he or she can rely on? Is a welfare commissioner involved with the patient? Does the patient use day services? To know such things are important. (E -87) - It is important to deepen understanding of patients with multimorbidity, including its management. (D -71) - Medical students tend to understand patients from a biomedical perspective. It is important to link the aspect with the life of the patient. (F -32) |
| 4. Communication skills |
| - Physicians need the skill that they briefly explain the condition to their patient.... While dialects can be a barrier to working in the community, communication with patients and their families is important.... (D -12). |
| 5. Team-based care |
| - It is important for physicians to know their own roles in the community. In areas beyond this, the ability to appropriately refer their patients to other physicians and interprofessional collaborations are also important. (C -41) - .... The ability to solve problems in cooperation with not only patients but also all health care professionals, community inhabitants, and the local government is important. (E -55) |
| 6. Quality and safety management in medical care |
| - Since the number of physicians involving in community medicine is small, each physician bears a heavy responsibility. The ability to relieve mental stress is important. For example, running and shopping that can be done on an individual level. Other than that, we can hold drinking parties or participate in festivals in the community. (C -81) - .... It is important to consider the safety management of CCC. Because problems such as medical accidents and human relations troubles may occur.... (C -97) |
| 7. Medical care in society |
| - It is important for physicians to know their own roles in the community. In areas beyond this, the ability to appropriately refer their patients to other physicians and interprofessional collaborations are also important. (C -41) - It is important to understand resources and issues in the community. (A -21) - .... Physicians' leadership plays an important role in considering cooperation in the community. (D -28) - .... The community inhabitants have roles other than their occupations. It is important for physicians to understand the roles.... It enables us to understand the social connection between people.... (C -87) - .... In other words, to cooperate with communication skills, with specialists, healthcare professionals, and the local government is important. (E -39) - From the view of local government, it is important to work with physicians on health promotion, prevention and welfare. (F -75) |
| 8. Scientific inquiry |
| - In the community, the ability to do research may be needed so that physicians' efforts to improve care are not self-righteous. Like action research.... (C -73, C -74) - ..... It is important to know the community and to be able to carry out community diagnosis.... (E -89) |
| 9. Lifelong learning attitude |
| - Problem solving ability and self-learning ability are important. (E -43) - .....It is important to be able to analyze what has been experienced and learned in the process, not to judge only by the results..... (C -55) |
